# Point-of-Care Devices for Viral Detection: COVID-19 Pandemic and Beyond

**DOI:** 10.3390/mi14091744

**Published:** 2023-09-07

**Authors:** Sumit K. Yadav, Damini Verma, Ujala Yadav, Ashish Kalkal, Nivedita Priyadarshini, Ashutosh Kumar, Kuldeep Mahato

**Affiliations:** 1 Department of Biotechnology, Vinoba Bhave University, Hazaribagh 825301, Jharkhand, India; 2 Department of Biosciences and Bioengineering, Indian Institute of Technology Roorkee, Roorkee 247667, Uttarakhand, India; 3 Department of Life Sciences, Central University of Jharkhand, Ranchi 835205, Jharkhand, India; 4 Department of Zoology, DAV PG College Siwan, Jai Prakash University, Chhapra 841226, Bihar, India; 5 Department of Electrical Engineering, University of Notre Dame, Notre Dame, IN 46637, USA; 6 Department of Nanoengineering, University of California San Diego, 9500 Gilman Dr, La Jolla, San Diego, CA 92093, USA

**Keywords:** COVID-19, diagnostics, POC testing devices, SARS-CoV-2, viral sensor

## Abstract

The pandemic of COVID-19 and its widespread transmission have made us realize the importance of early, quick diagnostic tests for facilitating effective cure and management. The primary obstacles encountered were accurately distinguishing COVID-19 from other illnesses including the flu, common cold, etc. While the polymerase chain reaction technique is a robust technique for the determination of SARS-CoV-2 in patients of COVID-19, there arises a high demand for affordable, quick, user-friendly, and precise point-of-care (POC) diagnostic in therapeutic settings. The necessity for available tests with rapid outcomes spurred the advancement of POC tests that are characterized by speed, automation, and high precision and accuracy. Paper-based POC devices have gained increasing interest in recent years because of rapid, low-cost detection without requiring external instruments. At present, microfluidic paper-based analysis devices have garnered public attention and accelerated the development of such POCT for efficient multistep assays. In the current review, our focus will be on the fabrication of detection modules for SARS-CoV-2. Here, we have included a discussion on various strategies for the detection of viral moieties. The compilation of these strategies would offer comprehensive insight into the detection of the causative agent preparedness for future pandemics. We also provide a descriptive outline for paper-based diagnostic platforms, involving the determination mechanisms, as well as a commercial kit for COVID-19 as well as their outlook.

## 1. Introduction

**Figure 1:**
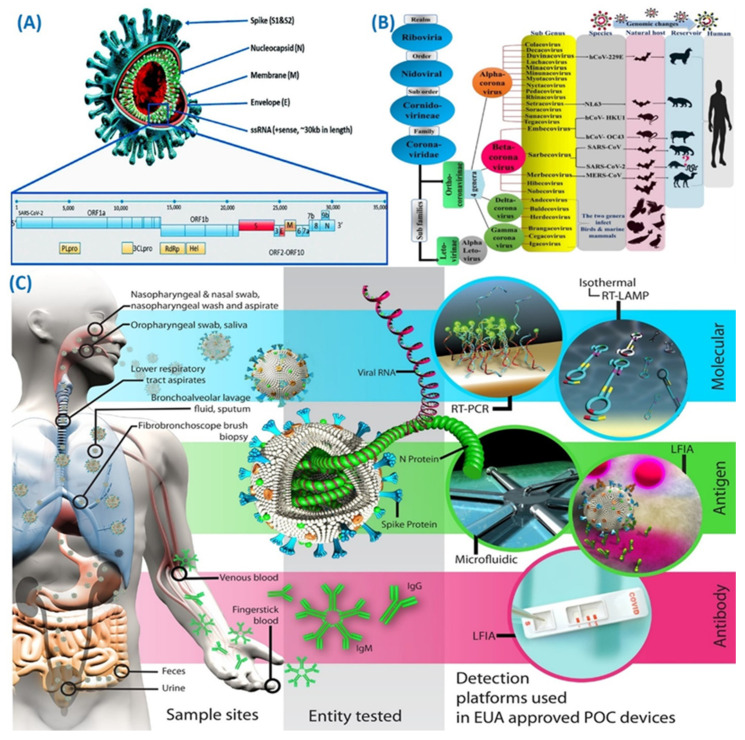
(**A**) Scheme depicting the structure of the SARS-CoV-2 virus (adapted with permission from Ref. [55], copyright 2020 MDPI). (**B**) Coronavirus taxonomy as per ICTV displaying the SARS-CoV-2 classification (adapted with permission from Ref. [56], copyright 2020 MDPI). (**C**) Human samples summary in which SARS-CoV-2 virus could be identified targets could be investigated, and detection platforms used in EUA-approved POC devices (adapted with permission from Ref. [48], copyright 2021 American Chemical Society).

The rapid global transmission and evolution of the novel coronavirus, known as SARS-CoV-2, leading to the outbreak of COVID-19 and subsequently declared as a global pandemic, have had profound effects on the lives of countless individuals worldwide [1,2]. This unprecedented situation has resulted in significant disruptions in healthcare, economy, and society [3]. Reflecting upon past events, previous outbreaks of infectious diseases like severe acute respiratory syndrome (recognized as SARS-CoV), as well as Middle East respiratory syndrome (recognized as MERS-CoV), have indeed caused notable social and economic consequences. However, these have not posed a comparable stage of danger to humanity as the COVID-19 pandemic [4,5]. Coronaviruses (CoV) are a type of enveloped virus characterized by their large size, positive-sense RNA genome, and non-segmented single-stranded RNA structure. Their genome length typically ranges from 26 to 32 kilobases [6,7] (Figure 1A). The classification of the taxonomy of coronaviruses (CoVs) has been performed by the International Committee on Taxonomy of Viruses (ICTV) recently, as part of the order Nidovirales, specifically within the family Coronavirideae. Within the subfamily of Coronavirinae, there are four major genera: Beta and Alpha coronaviruses that primarily cause infections in mammals, while Delta and Gamma coronaviruses mostly cause infections in birds. There are currently thousands of mutants of identified human CoV known to cause various infections such as respiratory, gastrointestinal, liver, and neurological diseases [8] (Figure 1B). Various SARS-CoV-2 mutations have arisen on a global scale, with certain strains displaying heightened transmissibility or a potential to resist antibodies. Notably concerning are variants like Alpha, Beta, Gamma, Delta, and Omicron, which have been linked to more severe clinical presentations [9]. The emergence of new strains of coronaviruses in humans seems to occur periodically, primarily due to the widespread prevalence of coronaviruses, the increase in human–animal interactions, their high genetic diversity, and the frequent genome recombination [10,11].

In recent times, coronaviruses (CoV) have emerged as significant pathogens responsible for the outbreak of respiratory diseases, including Middle East respiratory syndrome (MERS) in 2012 as well as severe acute respiratory syndrome (SARS) in 2002. A novel CoV called SARS-CoV-2 was recognized in cases of pneumonia in Wuhan, Hubei Province, China, in December 2019. This virus later became known as Coronavirus Disease 2019, i.e., COVID-19. In March 2020, the declaration of a pandemic, i.e., the COVID-19 outbreak, was announced by the World Health Organization (WHO). So far, COVID-19 has affected a staggering number of over 512 million individuals, resulting in approximately 6.2 million deaths [12,13,14]. While some drugs have received approval for COVID-19 treatment, there is currently no consensus on a widely applicable therapeutic regimen. Remdesivir is a type of antiviral medication designed to hinder the RNA-dependent RNA polymerase (RdRp) activity of the SARS-CoV-2 virus, thus impeding its ability to reproduce [15]. In response to the COVID-19 pandemic, the FDA has granted emergency use authorization for molnupiravir as a therapeutic option for COVID-19 patients. This drug also functions as an RdRp inhibitor, similar to Remdesivir [16]. Another pharmaceutical option against SARS-CoV-2, known as Paxlovid, combines two distinct drugs—nirmatrelvir and ritonavir—and has received FDA emergency use authorization. Nirmatrelvir, a peptidomimetic compound, serves as an inhibitor for the primary protease (Mpro) of SARS-CoV-2. This molecule covalently attaches to the catalytic cysteine (Cys145) residue on Mpro, preventing the virus from processing polyprotein precursors essential for replication [17]. Ritonavir, originally an inhibitor for HIV protease, plays a role in Paxlovid by extending the half-life of nirmatrelvir. Its mechanism involves inhibiting the metabolizing enzyme cytochrome P450 3A (CYP3A), thus enhancing the pharmacological effects of nirmatrelvir [18]. Vaccination efforts have made significant progress, but effectively managing COVID-19 patients involves initial diagnosis, instant isolation, and implementation of protective measures to avert further transmission [19,20].

The primary challenge in transmission of the SARS-CoV-2 virus is the identification of asymptomatic cases. Early diagnosis would act as a major part of preventing the virus transmission and controlling potential new waves of COVID-19. Additionally, prompt and early detection is vital for effective treatment during a pandemic, as it can greatly increase the prognosis of patients. Thus, there arises a continuous need to enhance existing diagnostic approaches and propose a sensitive, specific, and rapid methodology to determine SARS-CoV-2, particularly in point-of-care tests [21,22,23]. However, the current gold standard diagnostic approach for COVID-19 detection is conducted by the reverse transcription polymerase chain reaction known as RT-PCR that targets the SARS-CoV-2 RNA [24,25,26]. There is also increasing interest in loop-mediated isothermal amplification, i.e., LAMP techniques, like RT-LAMP [27,28,29,30,31]. The advancement of quick, POC molecular diagnostic experiments that exhibit comparable specificity and sensitivity to the existing gold conventional methods could effectively contribute to expanded testing capabilities [32,33].

Point-of-care testing, i.e., POCT, is a portable, user-friendly, and affordable technique that utilizes convenient and rapid analytical tools to provide immediate test outcomes at the step of sample collection [34]. POCT could be implemented in doctor’s offices, clinics, hospitals, or even homes by utilizing small sample volumes [35]. When compared to conventional laboratory testing [36], POCTs are formulated to identify distinct viral proteins or human antibodies linked to the SARS-CoV-2 virus. These tests offer relatively rapid results (typically spanning 15 to 30 min) and are economically more viable compared to RNA-based tests [37]. Furthermore, POCT assessments are conducive to self-administration at home by any individual. Deploying testing outside centralized test centers, particularly at urgent care or primary levels, could play a major part in prompt detecting and identifying COVID-19 cases, thus preventing community transmission. POC devices provide opportunities to (i) utilize more cost-effective and portable instruments, (ii) eliminate the requirement of sample transportation to testing laboratories for examination, (iii) decrease sample processing stages, (iv) utilize easily collectible samples such as anterior nasal swabs or saliva that did not need trained personnel for collecting the samples, and (v) record different entities (antibodies, antigen, virus) in asymptomatic or symptomatic patients, aiding in precise determination of individuals requiring clinical care or quarantine. While various types of POC devices have gained emergency use authorization (EUA) in different countries, there is ongoing validation of novel biosensing strategies and designs, as efforts continue to develop new devices with different techniques [38]. Currently, microfluidic paper-based analysis devices, i.e., using μPads have received enormous attention and have played a key role in advancing POCT. These analytical platforms incorporate injection [39], reaction [40], separation [41], and detection [42] functionalities within the paper by creating hydrophilic and hydrophobic channels. μPads offer numerous advantages, including minimal reagent consumption, excellent biocompatibility, ease of processing, simplicity in methodology, and low production cost. Notably, the development of μPads has experienced remarkable progress in current years [43].

Hence, considering the importance of determination techniques in POCT devices, it becomes crucial to examine and compare various present determination approaches on microfluidic paper-based analysis devices (μPads) and other POCT devices. In the current review, our emphasis lies on the advancements in POCT devices for SARS-CoV-2 determination. Initially, we provide an overview of the developed POCT methods for identification of SARS-CoV-2, encompassing clustered regularly interspaced short palindromic repeats or CRISPR-associated proteins (CRISPR/Cas), nucleic acid amplification tests (NAAT), and immunoassay tests systems. Additionally, we explore innovative POCT diagnostic platforms that integrate advanced technologies such as nanotechnology, aptamers, surface-enhanced Raman spectroscopy (SERS), and clustered regularly interspaced short palindromic repeats (CRISPR)-Cas. Lastly, we discussed the shortcomings and prospects for developing POCT analytical devices, while also providing insights into potential research opportunities and directions in this field. Although μPads are widely utilized due to their portability, there is still significant scope for their upgradation in terms of detection sensitivity and stability.

## 2. SARS-CoV-2 Diagnostic Targets and Point-of-Care Devices

Currently, there are three main categories of COVID-19 testing methods: antibody tests, antigen tests, and molecular diagnostics. The test of RNA diagnostics is employed to determine the SARS-CoV-2 RNA presence, while antigen tests aim to identify specific viral proteins. Conversely, antibody tests are utilized to ascertain the presence of antibodies developed by an individual against the virus [44]. In RNA tests, various RNA genome regions are targeted, while antigen examinations focus on structural proteins, i.e., antigens that exist within the viral envelope (Figure 1C). The SARS-CoV-2 virus possesses a single-stranded RNA having target genes like envelope genes, RNA-dependent RNA polymerase, S-protein, N-protein, ORF8, and ORF1b [35]. Among these, the four main structural proteins comprise the nucleocapsid protein (N) [45], matrix protein (M), small envelope protein (E), and spike surface made up of glycoprotein (S) [46].

The RT-PCR has become the gold conventional approach for the determination of the SARS-CoV-2 virus owing to their rapid amplification and high specificity capabilities. However, the thermal cycling requirements pose challenges when adapting this technology for portable devices, as precise temperature control is necessary to ensure accurate amplification of genetic material in the sample. Several RT-PCR-based POC devices have gained EUA. These include the Visby Medical COVID-19 POCT from Visby Medical (San Jose, CA, USA), the BioFire Respiratory Panel 2.1-EZ from BioFire Diagnostics, LLC (Salt Lake City, UT, USA), the cobas SARS-CoV-2 and Influenza A/B Nucleic Acid Test from Roche Molecular Systems, Inc. (Rotkreuz, Switzerland), the Accula SARS-CoV-2 Test from Mesa Biotech Inc. (San Diago, CA, USA), and Inc Xpert Xpress SARS-CoV-2 DoD, Xpert Xpress SARSCoV-2/Flu/RSV, Xpert Xpress SARS-CoV-2 from Cepheid, Sunnyvale, USA [47,48]. The SARS-CoV-2 determination of structural proteins in antigen tests heavily relies on the utilization of distinct monoclonal antibodies. These investigations have gained attention as a valuable element within a wide-ranging community testing approach aimed at minimizing the spread of the virus [49].

Antigen tests offer quicker results compared to PCR techniques, providing outcomes within minutes. However, they possess lower sensitivity due to the absence of target amplification. Several antigen EUA-approved point-of-care devices have demonstrated success, including the Status COVID-19/Flu by Princeton BioMeditech Corp, Sofia 2 Flu + SARS Antigen FIA (all 3 from Quidel Corporation), the QuickVue SARS Antigen Test, Sofia 2 SARS Antigen FIA, the Clip COVID Rapid Antigen Test by Luminostics, Inc., the BD Veritor System for Rapid Detection of SARS-CoV-2 by Becton, Dickinson and Company, LLC, the BinaxNOW COVID-19 Ag Card by Abbott Diagnostics Scarborough, Inc., the CareStart COVID-19 Antigen test by Access Bio, Inc., and LumiraDx SARS-CoV-2 Ag Test by LumiraDx UK Ltd. These techniques can detect nucleocapsid protein antigen qualitatively from SARS-CoV-2, which necessitates the extraction buffer inclusion for virus particle disruption in the specimen as well as responsible for exposure of the viral nucleoproteins present internally [47,48].

The certainty of protection against reinfection for patients who have recuperated from COVID-19 as well as possess antibodies remains uncertain due to reported instances of reinfection, as documented in both confirmed and suspected cases [48,50]. Similarly, individuals who have been recently infected may test positive for antibodies while still actively spreading the virus, depending on the sampling for serologic testing and timing of infection [51]. While over 100 serology tests have received EUA (including pending submissions), a limited number of POC devices got approved, such as the Sienna-Clarity COVIBLOCK COVID-19 IgG/IgM Rapid Test Cassette from Salofa Oy, the MidaSpot COVID-19 Antibody Combo Detection Kit from Nirmidas Biotech, Inc., the RapCov Rapid COVID-19 Test from Advaite, Inc., the RightSign COVID-19 IgG/IgM Rapid Test Cassette from Hangzhou Biotest Biotech, and the Assure COVID-19 IgG/IgM Rapid Test Device from Assure Tech [47,48].

As per the WHO rules, RT-PCR is considered the standard diagnostic method for SARS-CoV-2 determination owing to its specificity and high sensitivity. However, this conventional approach has certain drawbacks, including the requirement for skilled personnel and expensive laboratory equipment [52,53]. Moreover, accurate RNA extraction from the sample is essential before conducting the RT-qPCR test, requiring reliable equipment, reagents, and precautions to prevent contamination throughout the analysis and sampling process. On the other hand, antibody-based tests often require a minimal blood sample obtained through fingerstick or venipuncture, which can be challenging in underdeveloped areas [54]. Hence, paper-based POC diagnostic approaches have emerged as a potent complementary approach to overcome these limitations associated with conventional diagnostic methods.

## 3. Microfluidic-Based Smart Diagnostic Platform for COVID-19 Diagnosis

Microfluidics-based POC diagnostics are valuable tools for detecting SARS-CoV-2, offering integrated platforms through the combination of various techniques on microfluidic chips. These platforms, initially commercialized for analyzing and tracking oncological disease biomarkers, have now become a reality. The current pandemic has further uncovered the boundaries of conventional PCR-based techniques, which rely on skilled personnel and centralized laboratory setups. To overcome these challenges, microfluidics-based diagnostics have shown great potential [57,58]. Significant technological progress has occurred by incorporating microfluidic-based approaches for genetic material determination in recent years [59,60]. Successful demonstrations have been conducted, showcasing the detection of viruses, genetic material testing, and outcomes visualization using simple microfluidic-based methods [61,62]. These devices have been tested for genetic material detection in COVID-19 patients, exhibiting higher overall efficiency compared to assays such as LFA and reverse transcriptase LAMP [27,63].

**Figure 2:**
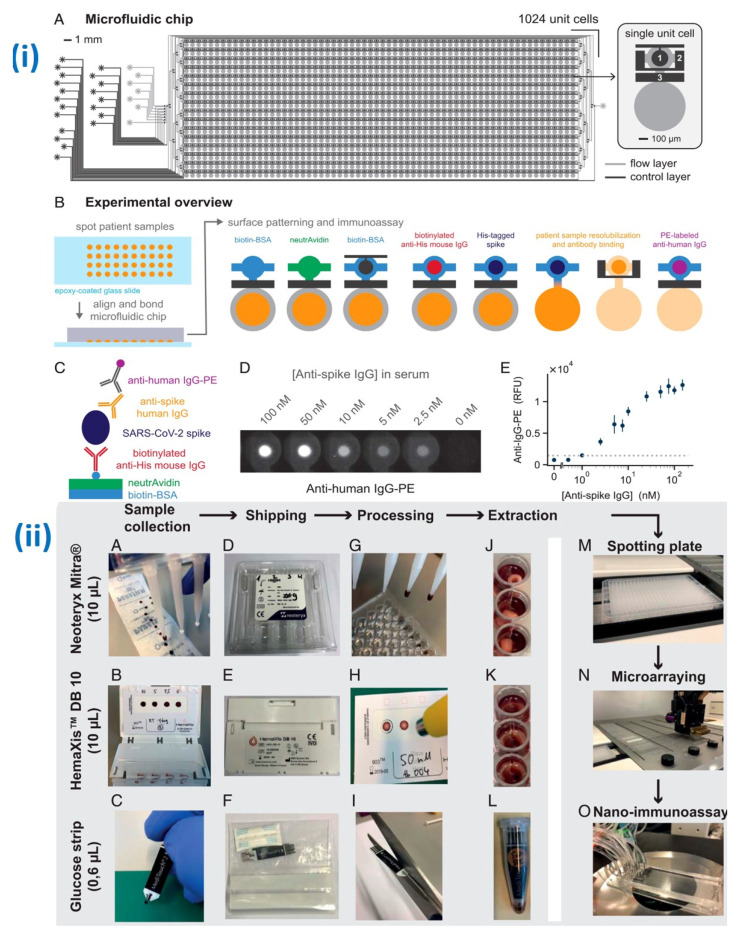
(**i**) (**A**) The microfluidic chip design comprises 1024-unit cells, enabling high-throughput detection of SARS-CoV-2. (**B**) The experimental process is depicted in an illustrative manner. (**C**) The chip facilitates the sandwich immunoassay process. (**D**) The fluorescence response of antihuman IgG-PE is observed for the anti-spike antibodies present in human serum. (**E**) An image displays the limit of detection (LOD) marked with a dashed line, along with the concentration of antihuman IgG-PE against anti-spike IgG. (**ii**) Schematic illustration of ultralow-volume whole blood sampling and processing developed by Swank et al. (**A**) The Mitra^®^ device and (**B**) the HemaXis™DB10 device are used to collect 10 μL of whole blood, while (**C**) the blood glucose test strip collects 0.6 μL of whole blood. After collection, (**D**–**F**) the blood samples are dried, allowing the devices to be shipped via regular mail under ambient conditions. Upon arrival at the laboratory, (**G**) the Mitra^®^ tips are removed and placed in a 96-well plate, (**H**) the HemaXis™DB10 cards are punched, and the filter discs are placed in a 96-well plate, and (**I**) the glucose test strip is cut to size and placed in an Eppendorf tube. Next, (**J**–**L**) the blood samples are extracted in a buffer solution through overnight incubation at 4 °C, followed by (**M**) transfer to a spotting plate. Subsequently, the samples are (**N**) microarrayed and (**O**) analyzed using the NIA device (adapted with permission from Ref. [68], copyright 2021 Proceedings of the National Academy of Sciences).

It is likely that SARS-CoV-2 will not be the final pandemic we have faced, highlighting the importance of technological preparedness and adaptability for future outbreaks. The need for high-throughput and rapid diagnostic strategies for SARS-CoV-2, in the form of portable POCT systems, remains urgent [64]. In recent years, miniaturized biosensors have emerged as promising analytical platforms because of their unique properties, rapid analysis, reliable specificity, high sensitivity, and consistent results [65,66,67]. As an example, Sawank and coworkers have developed a microfluidic nano immunoassay (NIA) called NIA for simultaneous anti-SARS-CoV-2 IgG detection on a single device in 1024 samples. The design of the microfluidic NIA device is shown in Figure 2(i) and demonstrates 100% specificity and 98% sensitivity [68]. Furthermore, the creation of an extensive serology platform that is affordable and easily accessible requires the exploration of alternative methods for venipuncture. To address this challenge, Swank et al. devised a sample collection and processing system that allows for non-invasive analysis using dried whole blood samples obtained through a convenient finger prick. The researchers conducted experiments utilizing three distinct approaches for collecting, shipping, processing, extracting, and analyzing dried whole blood samples, as illustrated in Figure 2(ii). They evaluated the performance of two commercially accessible devices designed for the collection of precise 10 μL quantities of whole blood: the Neoteryx Mitra^®^ and the DBS System SA HemaXis™DB10. In forthcoming times, the NIA generates the possibility for individuals to acquire a straightforward blood-sampling kit that comprises a lancet, a blood-sampling tool, and a pre-addressed return envelope. These kits could be obtained conveniently from local pharmacies or supermarkets (refer to Figure 2(ii)). The kit’s usage is uncomplicated and user-friendly within the confines of one’s personal space, where a minor finger prick is performed to collect the blood using the provided device. Subsequently, the device containing the blood sample can be dispatched via regular mail to a central laboratory without necessitating specialized biosafety precautions. This central laboratory would then conduct an analysis of the blood sample for one or more biomarkers, interpret the resulting data, and convey the test outcomes back to the individual through means such as smartphones, email, or regular mail.

**Figure 3:**
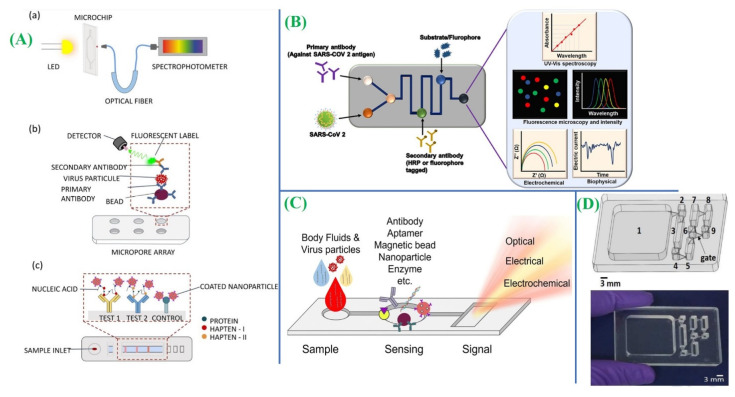
(**A**) Optical-based microfluidic platforms have been illustrated for the detection of respiratory viruses or their products using various techniques: (**a**) detection based on absorbance measurements, (**b**) fluorescent detection utilizing a micropore array, and (**c**) colorimetric detection employing a paper-based microfluidic platform with RT-LAMP (adapted with permission from Ref. [73], copyright 2021 Springer Nature). (**B**) Several microfluidics techniques have shown potential for the detection of SARS-CoV-2 using antibodies against the virus (adapted with permission from Ref. [69], copyright 2020 Frontiers). (**C**) Schematic illustration of an overview of several microfluidic techniques used for respiratory virus detection based on nanoparticles, aptamers, enzymes, antibodies, or magnetic beads (adapted with permission from Ref. [73], copyright 2021 Springer Nature). (**D**) The IFAST RT-LAMP device is designed with a sample chamber that is interconnected to wash chambers and a detection chamber through gates and a photograph of an IFAST RT-LAMP microfluidic device specifically developed for the detection of SARS-CoV-2 RNA (adapted with permission from Ref. [72], copyright 2021 Elsevier).

In a different analysis, a potential microfluidic technique has been proposed for antibody detection towards SARS-CoV-2, as depicted in Figure 3B. The integration capability of the microfluidic substrate with fluorescence, absorbance, and other diagnostic methods provides advantages over conventional diagnostic strategies, as illustrated in Figure 3A [69]. Significantly, the sensor device enables quantitative measures within a linear detection range of 585.4 copies/μL to 5.854 × 10^7^ copies/μL, having 231 copies/μL sensitivity. Yakoh et al. developed an electrochemical device for testing COVID-19 antibodies, delivering results within 30 min [70]. The utilization of a paper-based sensor presents an opportunity to transform the point-of-care testing (POCT) platform, providing the desired sensitivity and characteristics. This approach is particularly advantageous due to its cost-effectiveness, portability, and easy replacement options. Alafeef and his team employed a paper-based electrochemical technique to create detecting probes for genetic material testing [71]. Ganguli et al. engineered a portable RT-LAMP device designed to identify SARS-CoV-2 in clinical samples such as nasal swabs. Figure 3C presents a diagram illustrating the user-friendly and readily available point-of-care device. The device exhibited a detection limit of 50 RNA copies/μL and provided real-time detection of the viral genome within a 30-min timeframe.

The cost-effective and validated “SARS-CoV-2 RapidPlex” demonstrates a high level of sensitivity, enabling the simultaneous detection of multiple key biomarkers associated with the SARS-CoV-2 virus. These biomarkers include immune response, viral contamination, and disease severity, making it suitable for home-based diagnostics. In a study, Mateos et al. [72] developed an integrated on-chip platform that combines RNA extraction using immiscible filtration assisted by surface tension (IFAST) with colorimetric reverse transcription loop-mediated isothermal amplification (RT-LAMP) for RNA amplification and detection. The platform utilizes two sets of primers that target the open reading frame 1a (ORF1a) and nucleoprotein (N) genes of SARS-CoV-2 as shown in Figure 3D. Figure 3 showcases the extensive application of microfluidics across various domains, encompassing biomedical sensors, equipment, and even small valves. In recent years, nucleic acid techniques utilizing microfluidics, like CRISPR, LAMP, and RT-PCR, have been utilized to identify SARS-CoV-2. This section presents the up-to-date research findings from researchers as well as discusses the availability of POCT devices in the market.

### 3.1. LAMP Tests

**Figure 4:**
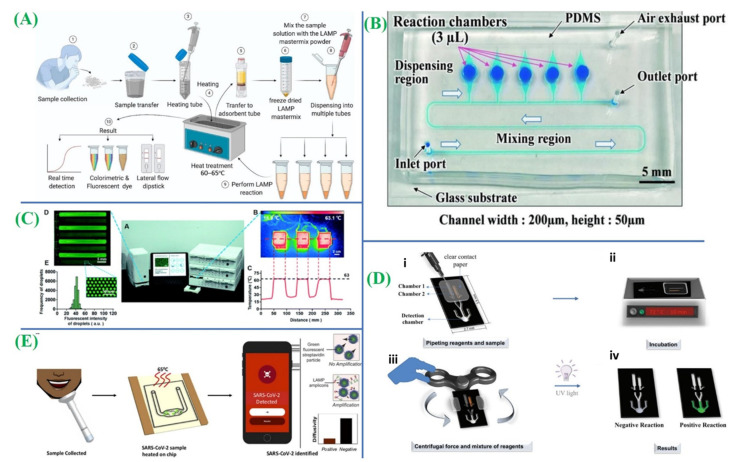
(**A**) A schematic diagram illustrating the process of LAMP detection (adapted with permission from Ref. [74], copyright 2020 MDPI). (**B**) The microfluidic chip is designed with a sequential fluid dispensing structure (adapted with permission from Ref. [75], copyright 2021 Royal Society of Chemistry). (**C**) Illustration of digital LAMP quantification having the da-Slip Chip by utilizing the random-access system (adapted with permission from Ref. [76], copyright 2021 Royal Society of Chemistry). (**D**) A schematic demonstration depicting the process of RT-LAMP amplification and detection in centrifugal PS-T microdevices: (**i**) introduction of reagents and sealing with transparent contact paper; (**ii**) incubation in a thermo block; (**iii**) centrifugation using a fidget spinner to rupture the valve; and (**iv**) visual detection under UV radiation (adapted with permission from Ref. [77], copyright 2021 Royal Society of Chemistry). (**E**) Development of a LAMP-integrated microfluidic chip and particle diffusometry (adapted with permission from Ref. [78], copyright 2022 Elsevier).

Isothermal amplification techniques, like loop-mediated isothermal amplification (LAMP), play a crucial role in isothermal amplification testing (IAT) and offer the advantage of detecting various targets simultaneously within a single reaction. Several studies have demonstrated that LAMP exhibits higher sensitivity and accuracy compared to RT-PCR for SARS-CoV-2 detection. The LAMP process is depicted in Figure 4A [74], involving the amplification of viral nucleic acids and LAMP-specific primers extracted at a constant 60–65 °C temperature. The outcomes are typically generated through fluorescence or colorimetry, which could be seen by a compact device or the naked eye. The detection and interpretation of LAMP results do not rely on bulky instruments, making it suitable for community and household self-screening.

Natsuhara and colleagues introduced a microfluidic chip with dispensing and mixing sections capable of continuous fluid dispensing (Figure 4B) [75]. This chip enables the analysis of communicable diseases like influenza and COVID-19 through LAMP-based colorimetry within individual chambers. Lyu and co-authors devised a droplet array slip-chip for high-throughput COVID-19 determination as shown in Figure 4C [76]. The facile movement of fluid was achieved by chip sliding which can avoid the precise bonding challenges associated with conventional chips featuring high-precision microchannels. In a study by de Oliveira and co-workers, a manually controlled centrifuge microfluidic device was employed for LAMP-based SARS-CoV-2 detection, utilizing a fidget spinner (Figure 4D) [77]. Although this device is portable and independent of a specific instrument, it exhibits inaccurate speed control, which may lead to errors if not operated properly. While the aforementioned works primarily focus on designing microfluidic chip structures for fluid control, there arises a requirement for a portable signal readout device. Smartphones have emerged as reliable signal readout devices for LAMP, offering improved computing power and imaging capabilities. Colbert and co-workers proposed a technique for SARS-CoV-2 identification by merging LAMP with particle diffusometry as shown in Figure 4E [78]. The smartphone is utilized for capturing images of samples comprising fluorescent beads after RT-LAMP, facilitating the detection process.

### 3.2. RT-PCR Tests

**Figure 5:**
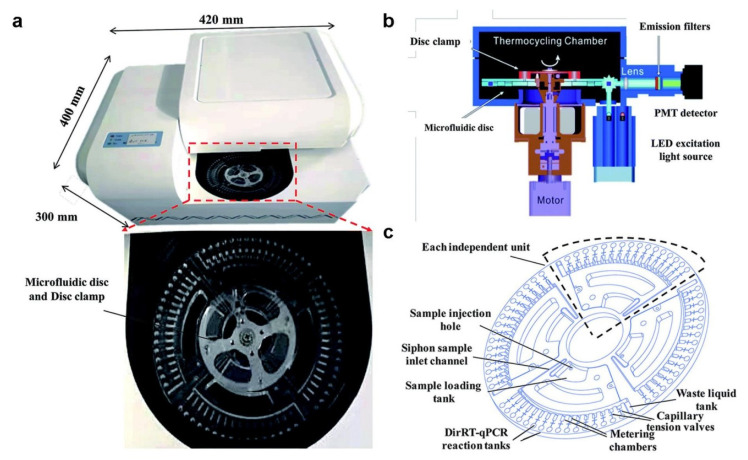
(**a**) A photograph showcasing a compact and portable centrifugal microfluidic device; (**b**) a schematic diagram illustrating the different components of the microfluidic device; and (**c**) a schematic illustration highlighting the structure and functional regions of the centrifugal microfluidic chip (adapted with permission from Ref. [83], copyright 2020 Royal Society of Chemistry).

The RT-PCR technique integrates complementary DNA (cDNA) PCR and RNA reverse transcription technologies. By leveraging mass and heat transfer methods based on fundamental hydrodynamic principles, microfluidics has the potential to enhance detection accuracy and reduce the time required for RT-PCR, particularly in terms of the necessary temperature variations. Incorporating microfluidic devices into RT-PCR testing can lead to more compact and rapid processes. Turiello and co-authors introduced an automated, rotationally driven microfluidic platform designed for the purification and enrichment of SARS-CoV-2 RNA [79]. The isolation of the virus for sample enrichment in the device is achieved using nanotrap magnetic particles, which effectively remove complex matrices and prevent the inhibition of RNA amplification and detection. The utilization of portable and microfluidic biochips determination devices that employ the RT-PCR technique greatly enhances the speed and accuracy of SARS-CoV-2 detection. Centrifugal microfluidic chips have found extensive application in disease diagnosis [80,81]. Furthermore, these chips enable highly automated and integrated multiple determinations, thereby enhancing practicality and functionality [82]. Figure 5 illustrates a direct RT-PCR approach proposed by Ji and his group, utilizing a centrifugal microfluidic chip device [83]. Centrifugal microfluidic systems are widely employed for nucleic acid detection due to their simplicity, self-contained fluid control, and minimal environmental contamination risks. One notable advantage of microfluidic chips is their capability to detect multiple targets simultaneously. The differentiation between COVID-19 and influenza, which exhibit similar symptoms, can enable widespread early screening and alleviate the burden on healthcare systems [84,85,86]. However, inconsistent sampling practices and sample contamination can result in inaccurate test outcomes [87]. These challenges hinder the widespread RT-PCR usage. We trust that standardizing and automating the sampling process within microfluidic chips can address this issue.

### 3.3. CRISPR-Associated Proteins System (Cas) Tests

**Figure 6:**
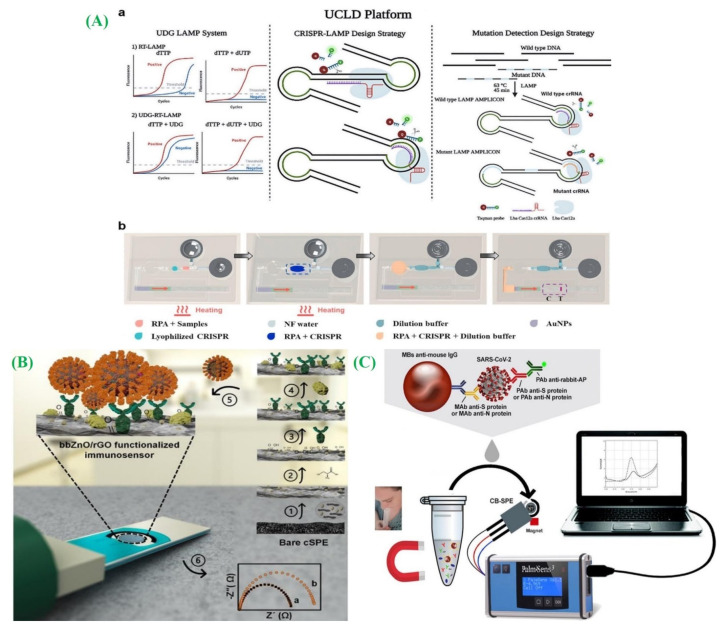
(**A**) An overview of the CRISPR–LAMP detection platform, designed to offer universal stability and precise results (**a**) (adapted with permission from Ref. [89], copyright 2020 American Chemical Society), and a microfluidic device utilizing lateral flow technology, which operates independently without the need for external devices, and incorporates a hand-warmer pouch as its power source (**b**) (adapted with permission from Ref. [92], copyright 2022 Elsevier); (**B**) a graphical representation depicting the sequential process of immuno-biosensor preparation (adapted with permission from Ref. [101], copyright 2020 American Chemical Society); and (**C**) a schematic demonstration of the detection process of electrochemical biosensor for a saliva sample (adapted with permission from Ref. [103], copyright 2020 Elsevier).

CRISPR, recognized as a powerful gene-editing tool, has been referred to as “molecular scissors” [88]. Zhang et al. devised a system that combines CRISPR with fluorophore-quencher DNA probes for signal amplification and detection purposes (Figure 6A(a)) [89]. Ramachandran et al. implemented isotachophoresis (ITP) on a microfluidic chip in combination with CRISPR and LAMP, enabling the diagnosis of COVID-19 within a 35-min timeframe [90]. In this system, Cas12, along with the guide DNA, selectively binds to the target DNA, leading to fluorophore–quencher DNA probe cleavage. However, the efficiency of CRISPR is hindered by time-consuming amplification and nucleic acid processes as well as the dependence on huge instruments for fluorescent signal readout. Silva and co-authors developed a catalase-mediated assay for CRISPR-based detection of SARS-CoV-2 [91]. Certain scientists have devised testing techniques that operate autonomously without the need for external apparatus. Li and his group introduced a lateral flow microfluidic device that utilizes a hand-warmer pouch serving as a heat source (Figure 6A(b)) [92]. In this approach, the reagent’s freeze-dried powder is preloaded into a reaction chamber. Then, the handler can manually manipulate the liquid as well as observe the outcomes with the bare eye. While numerous RNA detection methods that are both highly sensitive and suitable for POC application exist, they are marred by a susceptibility to non-specific amplification when conducted under isothermal conditions. This susceptibility consequently results in inaccurate positive outcomes. To counteract this concern, the incorporation of isothermal amplification techniques with CRISPR technology has proven to be instrumental in mitigating the likelihood of non-specific detection [93,94]. Leveraging these attributes, a multitude of approaches that combine CRISPR technology with isothermal amplification have emerged. These innovative techniques effectively enhance the amplification of target genes and consequently elevate the overall efficacy of COVID-19 detection procedures.

### 3.4. Electrochemical Biosensors

Electrochemical biosensors have received enormous attention owing to their simplicity, affordability, and potential for miniaturization. These tiny devices utilize tailored electrodes that serve as receptors or transducers, depending on the specific requirements, to enable real-time, specific, and accurate target monitoring [95,96,97]. By extracting potentiometric or amperometric signals from the sensing electrode, information linked to the analyte presence can be obtained [98,99,100].

In their study, Sanati-Nezhad and his group fabricated an electrochemical immunosensor approach to determine the nucleocapsid protein antigens of SARS-CoV-2. They utilized a bbZnO/rGO nanocomposite coating on carbon screen-printed electrodes (SPEs) to enhance antibody adsorption (Figure 6B) [101]. The resulting device system was connected to a readout system, which generated electrochemical signals in COVID-19 presence. Similarly, Ali et al. employed improved 3D printing technology to construct a 3D reduced-graphene-oxide (rGO) electrode and integrated it with a microfluidic device, serving as an electrochemical sensor [102]. This setup allowed for the antibodies specific determination towards SARS-CoV-2, achieving a 2.8 × 10^−15^ M limit of detection (LOD). Additionally, Fabiani and the group developed a miniaturized electrochemical sensor utilizing magnetic beads and a carbon black-based electrode for SARS-CoV-2 determination (Figure 6C) [103]. The utilization of magnetic beads offers advantages in terms of preconcentration, reduced washing steps, and enhanced sensitivity and reliability. Additionally, the application of an external magnetic field enables the removal of interference from seasonal H1N1 influenza virus during detection. Zhao et al. demonstrated the feasibility of using a smartphone-based electrochemical sensor for the determination of SARS-CoV-2 RNA, eliminating the necessity for extensive laboratory processes and equipment [104]. This “plug-and-play” setup opens up possibilities for portable testing. Another recent study integrated the electrochemical platform with a wireless module, facilitating the development of graphene electrodes for rapid COVID-19 identification [105]. The “SARSCoV-2 RapidPlex” test, which is cost-effective and highly sensitive, can determine SARS-CoV disease and offer information on COVID-19 key aspects (disease severity, immune response, and viral infection). This test has the efficiency to be performed at home, offering convenience and accessibility to individuals.

## 4. COVID-19 Diagnosis Using Paper-Based Diagnostic Platform

Paper-based analytical systems offer significant potential for bringing POC diagnostic systems to developing regions, primarily owing to their exceptional characteristics including transportation, convenient storage, eco-friendliness, chemical inertness, flexibility, ease of modification, porosity, and biocompatibility [106,107]. Furthermore, these devices allow for the application of various sample types, and sample transfer can be achieved without the need for additional power, relying on capillary forces. Over the last three decades, paper-based POC tests have been proposed for diverse biomedical uses and have been introduced as both “over-the-counter” products, like “professional market” products, pregnancy testing, and glucose monitoring capable of diagnosing various cancers, hemopathies, lipidoses, diabetes, cardiac markers, and infectious diseases. Notably, paper-based POC devices have shown a key role in addressing the current COVID-19 pandemic. The platforms for paper-based POC diagnostics range from simple 1D formats like lateral flow assays (LFA) and dipsticks to more complex 3D platforms, including electrochemical paper-based assay devices (ePAD) and microfluidic paper-based assay devices (μPAD).

Microfluidic paper-based devices (μPads) deliver several benefits, including simplicity, ease of processing, low production cost, good biocompatibility, and minimal reagent consumption. Consequently, there has been an enormous rise in the development of μPads in recent times [43]. Various methods have been employed to fabricate μPads, like stereoscopic printing [108], flexographic printing [109], one-step plotting technology [110], cutting [111], plasma processing [112], laser processing [113], wax printing [114], inkjet printing [115], and photoetching [116,117]. Amongst these approaches, LFA stands out as a well-established paper-based diagnostic method that has gained significant consideration and investment from manufacturers and researchers for COVID-19 diagnostic kit development. A characteristic LFA consists of an absorbent pad, a nitrocellulose membrane, a conjugate pad, and a sample pad. The initiation of sample flow occurs from the sample pad as well as encounters the dried signal molecules onto the conjugate pad. The whole SARS-CoV-2 RNA, antibodies, antigens, and biomarkers necessary for SARS-CoV-2 detection could be used for the LFA platform.

### 4.1. LFA-Based Diagnostic Platforms

#### 4.1.1. For Viral Antigens

In contrast to RT-qPCR, antigen diagnostics offer a direct means of detecting SARS-CoV-2 as well as its associated proteins in a sample obtained from a nasal passage or nasopharyngeal swab, eliminating the need for sample pretreatment and amplification [118]. This approach enables faster, easier, and more cost-effective diagnosis of COVID-19 compared to RT-qPCR. Antigen diagnosis test relies on immunoassay reactions which include the interaction between antibodies and antigens. Despite the development of numerous paper-based antigen diagnostics, the rapid antigen tests’ sensitivity remains uncertain as well as low in comparison to RT-qPCR. The detection limit for antigen tests is around 10^5^ copies/mL, whereas RT-qPCR can detect levels even lower than 10^2^ copies/mL [119,120]. False-negative outcomes might result in lower sensitivity than antigen tests’ sensitivity when the target antigen’s concentration in the experimental specimen is decreased. To address these limitations, several studies have been conducted. Liu and the group introduced an innovative nanozyme-based chemiluminescence paper assay for the detection of SARS-CoV-2 S antigen. This approach integrates chemiluminescent immunoassays with LFA and nanozyme (Co-Fe@hemin-peroxidase), achieving a (360 TCID_50_/mL) sensitivity compared to ELISA [121].

**Figure 7:**
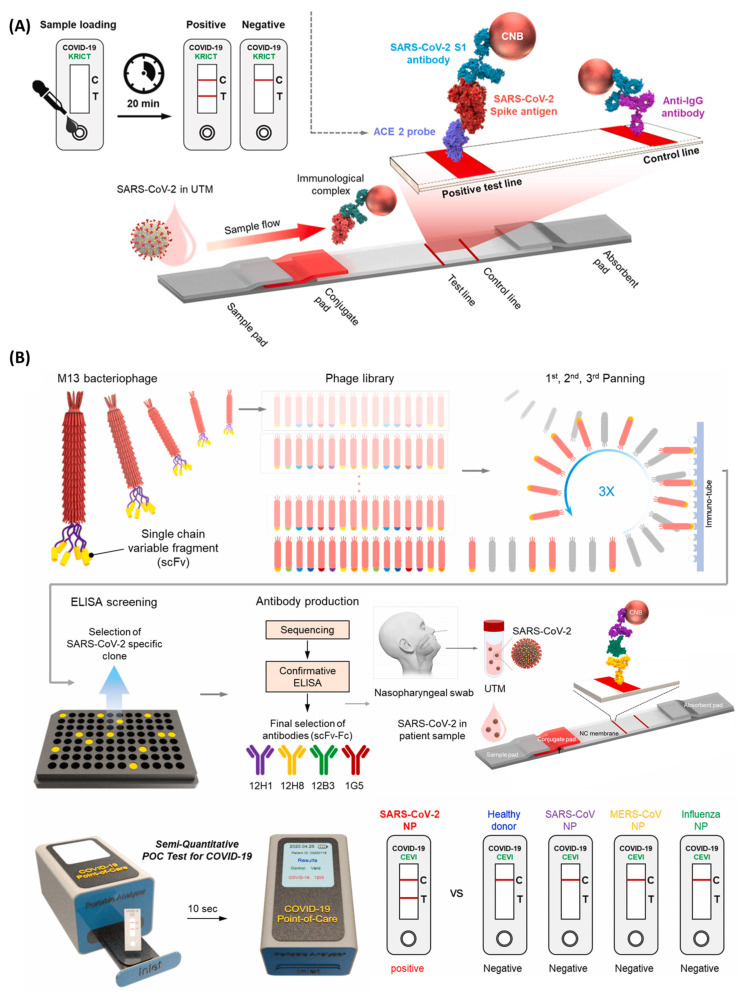
(**A**) Cellular receptor (ACE2)-based LFA for detecting SARS-CoV-2 S1 antigen (adapted with permission from Ref. [122], copyright 2021 Elsevier). (**B**) Development of highly specific and sensitive scFv-Fc fusion proteins (rapidly screened by phage display technology) based LFA for detection of the SARS-CoV-2 N protein (adapted with permission from Ref. [124], copyright 2021 Elsevier).

Furthermore, Kim and the group introduced an innovative method to quickly monitor SARS-CoV-2 S antigen. By utilizing angiotensin-converting enzyme 2 (ACE2), the cellular receptor for SARS-CoV-2, they effectively determined the SARS-CoV-2 S1 antigen from COVID-19 patients extracted clinical specimens (Figure 7A) [122,123]. Additionally, they developed fusion antibodies comprising crystallizable fragments (Fc) and single-chain variable fragments (scFv) specific to the SARS-CoV-2 N antigen employing phage display technology. These scFv-Fc antibodies were then applied to the LFA platform (Figure 7B) [124]. The quick scFv-Fc-based diagnostic kit exhibited good specificity, capable of distinguishing even the SARS-CoV N protein.

#### 4.1.2. For Viral Antibodies

**Figure 8:**
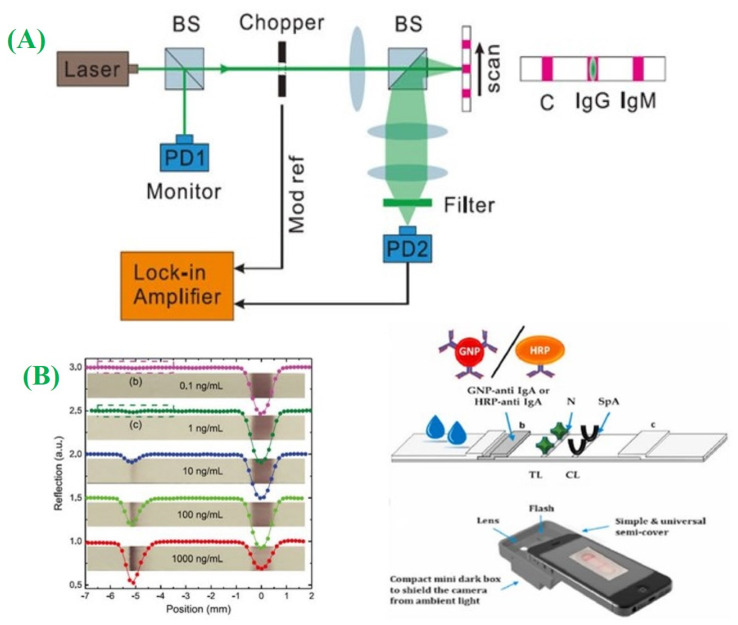
(**A**) The detection system is configured to quantify the results of the LFA using a photon-counting approach for the detection of IgG antibodies (adapted with permission from Ref. [129], copyright 2020 American Institute of Physics); and (**B**) the LFA strip designed to detect anti-SARS-CoV-2 IgA antibodies and a simple and universally compatible smartphone reader is employed to detect the optical signal emitted from the LFA (adapted with permission from Ref. [130], copyright 2021 Elsevier).

LFA enables the antibodies qualitative determination in serum samples. When SARSCoV-2 infects the human body, the immune system is activated to combat the virus. As part of this immune response, various immunoglobulins, which include IgM, IgG, and IgA, are generated to neutralize the virus and provide protection against future infections [125,126,127]. IgM antibodies typically emerge in the blood a few days following infection and become identifiable around 5 to 10 days after the symptom’s onset. In serological LFA, test lines (T1, T2) are formed by immobilizing antihuman antibodies of IgG (or IgM) on a nitrocellulose membrane, while a control line is created utilizing secondary antibodies produced in different hosts like mice and rabbits. When a sample comprising SARS-CoV-2-specific IgG or IgM antibodies is applied to the strip of LFA, the antibodies attached to gold nanoparticles conjugated with the S (or N) protein flow together as well as eventually attach to the test line, leading to a prominent color change. Zeng and the group introduced a lateral flow technique combing an IgG–IgM immunochromatographic assay, demonstrating higher sensitivity (85.29%) compared to individual IgM (82.35%) and IgG (61.76%) tests [128]. Peng and the group implemented a photon-counting technique to detect LFA and enhance sensitivity (Figure 8A). They utilized laser optical analysis to record SARS-CoV-2 antibody density in a straightforward manner [129]. Moreover, Roda and the group introduced a dual optical/chemiluminescence format for LFA, aiming for highly sensitive and affordable detection (Figure 8B) [130].

#### 4.1.3. For Viral RNA

**Figure 9:**
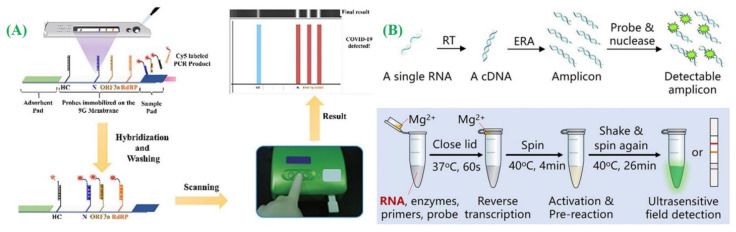
(**A**) The lateral flow strip membranes (LFSM) for the highly specific and sensitive method for detecting SARS-CoV-2 that enable simultaneous detection of multiple regions of SARS-CoV-2 RNA in a single test (adapted with permission from Ref. [131,132], copyright 2020 American Chemical Society). (**B**) The reverse transcription-enzymatic recombinase amplification (RT-ERA) operates on the principle of ultrasensitive, field-deployable, and simultaneous dual-gene detection of SARS-CoV-2 RNA (adapted with permission from Ref. [134], copyright 2020 Springer Nature).

Isothermal nucleic acid amplification is a viable approach that enables amplification at a consistent temperature and eliminates the requirement for bulky equipment like thermocyclers [45]. This technique offers advantages such as high sensitivity, specificity, convenience, and cost-effectiveness. A novel LFA was created as a potential substitute for conventional RT-qPCR, enabling the concurrent identification of genes of SARS-CoV-2, namely, N, ORF3a, and RdRp genes [131]. The product of PCR was generated through RT-PCR, thereafter a 30 min LFA analysis was conducted at 25 °C, with an LOD of 10 copies/test for a single gene (Figure 9B). Nevertheless, the overall assay duration still amounts to around 2 h, including the 100 min required for the PCR reaction. The incorporation of this technique into an LFA offers notable benefits like affordability, convenience, specificity, and high sensitivity, thereby facilitating the development of POC kits for detecting SARS-CoV-2 RNA (Figure 7A). Zhu and the group proposed a diagnostic approach utilizing multiplex reverse transcription loop-mediated isothermal amplification (mRT-LAMP) combined with LFA for COVID-19 diagnosis [132]. The whole analysis time period for this test is 1 h. A qualitative experiment was also performed that combined the LFA coupled with recombinase polymerase amplification (RPA) (Figure 9A) [133]. This test targeted the SARS-CoV-2 N gene and demonstrated the ability to detect SARS-CoV-2 N gene-comprising plasmid as few as 0.25–2.5 copies/μL. Xia et al. introduced a highly sensitive field-deployable method (detecting single-copy levels) for identifying the SARS-CoV-2 gene through the implementation of reverse transcription-enzymatic recombinase amplification (RT-ERA) (Figure 9B) [134].

### 4.2. Microfluidic and Electrochemical Paper-Based (μPADs and ePADs) Diagnostics Platforms

**Figure 10:**
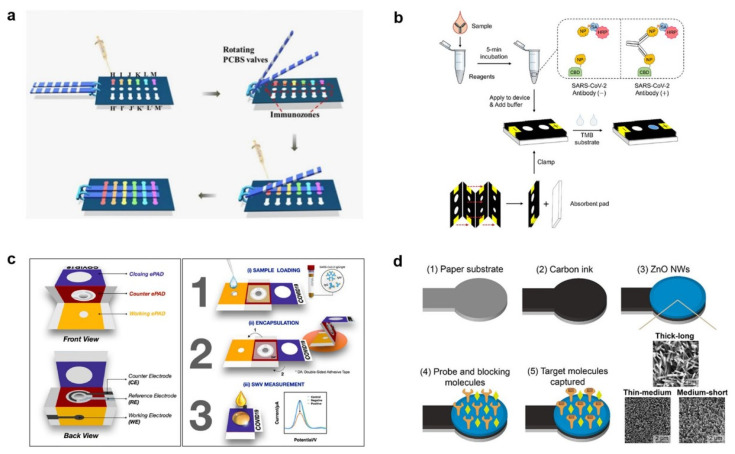
(**a**) PFST-μPADs for quantitative SARS-CoV-2 IgA/IgM/IgG assay (adapted with permission from Ref. [136], copyright 2021 American Chemical Society). (**b**) 3D μPADs are utilized for the detection of SARS-CoV-2 specific antibodies by leveraging the affinity between cellulose and the cellulose binding domain (adapted with permission from Ref. [139], copyright 2021 American Chemical Society). (**c**) A label-free ePAD for detecting SARS-CoV-2-specifc IgG and IgM antibodies (adapted with permission from Ref. [70], copyright 2021 Elsevier). (**d**) A novel ePAD proposed for COVID-19 diagnosis, incorporating a working electrode enhanced by ZnO nanowires (adapted with permission from Ref. [142], copyright 2021 Elsevier).

Typical LFAs provide a comfortable, speedy, and cost-effective option for testing for COVID-19. However, there is an ongoing debate regarding their sensitivity and specificity [135]. Moreover, their limitations in offering quantitative analyte results hinder their use in clinical practice [136]. To address these issues, μPADs have emerged as an appealing diagnostic kit that links the benefits of paper-based biosensing platforms with traditional microfluidic devices. Through various straightforward patterning approaches like PDMS printing, screen printing, wax printing, inkjet etching, and photolithography, it becomes effortless to create hydrophilic channels and hydrophobic barriers, enabling control over flow within the µPADs [61]. In contrast to conventional LFAs, μPADs offer the flexibility to direct flow in multiple directions (both horizontally and vertically) based on their design, enabling the quantitative determination of different analytes within a single device. Additionally, when compared to conventional microfluidic devices, μPADs possess inherent benefits like good biocompatibility, independence from external power sources, simplicity in fabrication, and cost-effectiveness [137]. As a result, μPADs are increasingly finding applications in various fields, including the biochemical industry, medical diagnosis, environmental monitoring, and POC diagnostics [137,138]. An example of their application is demonstrated by Gong et al., who introduced an instrument-free paper-based microfluidic enzyme-linked immunosorbent assay (ELISA) for quantitatively measuring IgG/IgM/IgA antibodies specific to SARS-CoV-2. By employing a pulling-force spinning top (PFST) in combination with a paper-based microfluidic approach (Figure 10a), they achieved blood–serum separation and detection of the antibodies [136]. Another group utilized cellulose as a substitute membrane material as well as employed a double-antigen sandwich format (Figure 10b) for a SARS-CoV-2 antibody test [139]. They constructed a 3D channel and fabricated a device to maintain a continuous flow rate. Additionally, Garneret et al. developed μPADs for SARS-CoV-2 RNA determination. They presented a user-friendly portable device that integrates paper microfluidics with isothermal nucleic acid amplification [140].

In recent developments, an electrochemical paper-based analytical device (ePAD) has emerged as a solution to enable quantitative data acquisition and enhance detection sensitivity. Unlike most μPADs that depend on optical readout through agglomeration of nanoparticles, often imposes limitations on the performance of the sensor [141]. Yakoh and group introduced a label-free ePAD specifically designed for antibodies IgM and IgG SARS-CoV-2-specific determination. This ePAD consists of three components: a closing ePAD, a counter ePAD, and a working ePAD (Figure 10c) [70]. Remarkably, this ePAD demonstrates high sensitivity (100%) in detecting targeted antibodies in clinical sera and could be explored for antigen determination as well. In other research, a novel ePAD-based platform for COVID-19 diagnosis was introduced that incorporates zinc oxide nanowires (ZnO NWs) grown directly on working electrodes (Figure 10d) [142]. By optimizing the ZnO NWs-enhanced working electrode, they determined SARS-CoV-2-specific antibody (CR3022) successfully even at 10 ng/mL, the lowest concentration in human serum.

## 5. Summary and Conclusions

Nearly three years have passed since the initial recorded case of COVID-19. Regrettably, due to the virus’s highly transmissible nature and the frequent emergence of new variant mutations, effective treatments for the virus are still unavailable. Consequently, mass detection, timely diagnosis, and physical interventions like social distancing remain crucial in curtailing the transmission of the virus. Hence, there is an ongoing need for the development of specific, highly sensitive, robust, and rapid POC diagnostic tests. In light of these requirements, this review aims to provide an overview of the existing diagnostic techniques used during the SARS-CoV-2 pandemic, focusing on their impact on public health and emergency response. The gold conventional tool for SARS-CoV-2 virus diagnosis is the detection of nucleic acid through RT-PCR. However, this diagnostic method involves time-consuming RNA extraction, taking approximately 3–4 h, and requires qualified technicians and expensive laboratory facilities. Although RT-PCR serves as the primary defense against the outbreak, different serological diagnostic approaches are also accessible to counterpart it. Nevertheless, cross-reactivity and the potential for false-positive results pose significant concerns. As the disease continues to spread, the immediate necessity lies in more affordable point-of-care tools.

The advancement of health monitoring systems has been greatly facilitated by the adoption of microfluidics technology and biosensors, enabling rapid detection methods. Microfluidic technology allows for the integration of complex analytical processes within small volumes, encompassing tasks such as sampling, mixing, separation, enrichment, washing, and temperature control. By leveraging these capabilities, microfluidics provides a cost-effective platform for the determination of SARS-CoV-2 that is rapid, accurate, and automated. Researchers have successfully incorporated different nucleic acid amplification approaches, involving CRISPR, LAMP, and RT-PCR, into microfluidic chips for diagnostic applications. Additionally, extremely sensitive analysis approaches like electrochemical biosensors, paper-based tests, and fluorescence-assisted tests have gained widespread usage. While these devices have demonstrated impressive performance in laboratory settings, there is still a considerable gap to bridge for commercial deployment. The operational requirements of commercial equipment often compromise the reliability of test results and render the products unaffordable for most individuals. We anticipate that with the increasing demand for testing, more exemplary methods will emerge in the market. In practical POC applications, instrument-independent determination is endorsed, particularly in resource-constrained environments. Therefore, downsizing analytical instruments becomes crucial to enhance mobility and energy efficiency, facilitating their suitability for field use. Additionally, transferring computer-based data processing to portable terminals would aid in the commercialization efforts [143]. Notably, current portable commercially available quantitative tools like pressure meters and glucometers have already been leveraged for analyte identification indirectly, representing the significant potential for the commercialization of instrument-free integrated quantitative nucleic acid detection [144,145].

Furthermore, this review offers an outline of current advancements in paper-based determination techniques for quick SARS-CoV-2 diagnosis. Paper, being easily accessible and inexpensive, allows for relatively low-cost mass production of biosensors. Moreover, paper’s inherent capability to facilitate fluid movement via capillary flow reduces the requirement for external pumps or equipment, making paper-based diagnostic systems highly attractive in the market. Several cost-effective and rapid POC tests, including LFA-based diagnostic kits, have been proposed as powerful techniques to combat the outbreak of COVID-19. These tests have played a significant role in promptly identifying innovative infections and implementing quarantine records. While LFA-based tests have been used to complement the current gold standard technique, RT-qPCR, paper-based analytical devices have the efficiency for providing POC diagnostic devices for developing countries as well as developing it for future disease outbreaks beyond COVID-19. Despite the progress made in novel research on paper-based tests for reliable POC diagnostics, their application in COVID-19 diagnosis still faces challenges. One area of research focus is reagent storage, aiming to achieve stable storage of reagents within paper devices for instant use. Another important aspect is sample preparation, especially crucial for CRISPR and nucleic acids tests that often rely on amplification to improve the determination’s sensitivity. Additionally, multiplex detection is essential for many medical diagnostic tests due to the similarities in symptoms among different infectious diseases, like the common cold and SARS-CoV-2. Furthermore, certain diseases are co-existing due to multiple serotypes and pathogens [146]. Therefore, rapid and simultaneous multiple target detection, in comparison to single target, would greatly benefit medical diagnoses. Moreover, the introduction of artificial intelligence (AI) is expected to improve diagnostic performance. AI-based COVID-19 diagnosis has been progressively explored in lung determination imaging, including chest radiograph (X-ray) imaging and computed tomography (CT) [147,148] and chest radiograph (X-ray) imaging [149,150]. Through close collaboration between academia and industries, it is believed that a paper-based POC test with specificity and high sensitivity, while completely fulfilling the ASSURED standards, is within reach.

In the context of this review article, one particular study conducted by Li et al. demonstrated the highest sensitivity in detecting the CR3022 antibody, which is specific to the spike glycoprotein S1 of SARS-CoV-2, in human serum [142]. This antibody serves as a marker for COVID-19, with a remarkably low LOD of up to 0.4 pg mL^−1^. The study focused on enhancing the performance of paper-based ZnO-NW-enhanced Electrochemical Impedance Spectroscopy (EIS) biosensors through an experimental approach. In essence, this research contributes to the advancement of our knowledge regarding the effectiveness of nanomaterial-augmented EIS biosensing. Additionally, it provides insights into potential strategies for achieving even better EIS biosensing performance by manipulating the morphology of nanomaterials. The utilization of such high-performance paper-based nano biosensors holds significant promise for precise and swift diagnostic applications in effectively managing infectious disease crises.

## Data Availability

Not applicable.
